# RNA interference screen reveals a high proportion of mitochondrial proteins essential for correct cell cycle progress in *Trypanosoma brucei*

**DOI:** 10.1186/s12864-015-1505-5

**Published:** 2015-04-15

**Authors:** Diane-Ethna Mbang-Benet, Yvon Sterkers, Lucien Crobu, Amélie Sarrazin, Patrick Bastien, Michel Pagès

**Affiliations:** Université Montpellier 1, UFR Médecine, Laboratoire de Parasitologie-Mycologie, CHRU de Montpellier, 39, Avenue Charles Flahault, 34295 Montpellier, Cedex 5 France; CNRS 5290 - IRD 224 - Université Montpellier (UMR “MiVEGEC”), Montpellier, France; Département de Parasitologie-Mycologie, CHRU (Centre Hospitalier Universitaire de Montpellier), Montpellier, France; Montpellier RIO Imaging Facility, Montpellier BIOCAMPUS, UMS3426, Arnaud de Villeneuve Campus Imaging Facility - Institut de Génétique Humaine-CNRS, Montpellier, France

**Keywords:** *Trypanosoma brucei*, RNA interference, Cell cycle, Mitochondrion, Kinetoplast

## Abstract

**Background:**

Trypanosomatid parasites possess a single mitochondrion which is classically involved in the energetic metabolism of the cell, but also, in a much more original way, through its single and complex DNA (termed kinetoplast), in the correct progress of cell division. In order to identify proteins potentially involved in the cell cycle, we performed RNAi knockdowns of 101 genes encoding mitochondrial proteins using procyclic cells of *Trypanosoma brucei*.

**Results:**

A major cell growth reduction was observed in 10 cases and a moderate reduction in 29 other cases. These data are overall in agreement with those previously obtained by a case-by-case approach performed on chromosome 1 genes, and quantitatively with those obtained by “high-throughput phenotyping using parallel sequencing of RNA interference targets” (RIT-seq). Nevertheless, a detailed analysis revealed many qualitative discrepancies with the RIT-seq-based approach. Moreover, for 37 out of 39 mutants for which a moderate or severe growth defect was observed here, we noted abnormalities in the cell cycle progress, leading to increased proportions of abnormal cell cycle stages, such as cells containing more than 2 kinetoplasts (K) and/or more than 2 nuclei (N), and modified proportions of the normal phenotypes (1N1K, 1N2K and 2N2K).

**Conclusions:**

These data, together with the observation of other abnormal phenotypes, show that all the corresponding mitochondrial proteins are involved, directly or indirectly, in the correct progress or, less likely, in the regulation, of the cell cycle in *T. brucei*. They also show how post-genomics analyses performed on a case-by-case basis may yield discrepancies with global approaches.

**Electronic supplementary material:**

The online version of this article (doi:10.1186/s12864-015-1505-5) contains supplementary material, which is available to authorized users.

## Background

Trypanosomatids are flagellated protozoan parasites that belong to the Order of Kinetoplastida. Three of them are human pathogens: *Trypanosoma brucei*, responsible for the African human trypanosomiasis also known as sleeping sickness, *Trypanosoma cruzi*, the agent of Chagas’ disease in Latin America, and *Leishmania* sp., responsible for leishmaniases in many countries throughout the world. In addition to their importance in public health, the cell and molecular biology of these divergent eukaryotes is of great interest, and their study has revealed many original features. For example, the gene organization as large polycistronic-like clusters reminding that of prokaryotes is unique among Eukaryotes [1,2], and is thought to be related with the near-absence of RNA PolII promoters and transcriptional regulation [3]. The 26 Mb genome of *T. brucei* contains 9302–11100 predicted genes [4,5]. According to GeneDB [6], 4539 and 575 CDSs (Coding DNA Sequences) are annotated as encoding ‘hypothetical proteins, conserved’ and ‘hypothetical proteins, unlikely’, respectively. Several large studies using different approaches based on RNA interference (RNAi) have been reported with the aim of (i) giving clues on the function of the different CDSs in this parasite, (ii) finding regulators of the cell cycle progress, (iii) opening new avenues for drug design [7-10]. Two studies are of particular interest for the present study: (i) a case-by-case approach, in which almost all the CDSs of chromosome 1 were individually targeted by RNAi [7]; and (ii) a global approach with an 11× − coverage RNAi plasmid library [11] made of randomly sheared genomic DNA and cloned in a vector for the Tet-inducible expression of dsRNA [8].

Among many singularities, trypanosomatids possess a single mitochondrion containing a complex mitochondrial DNA organized in a dense network and termed kinetoplast. The kinetoplast is an essential organelle, not only because it contains a highly specialized form of mitochondrial DNA but also because its duplication and segregation are tightly associated to correct cell cycle progress, in particular cytokinesis [12-14]. The molecular mechanisms governing this link between cytokinesis and the segregation of the kinetoplast and the basal body of the single flagellum are slowly being elucidated, but much remains to be done [15,16]. The *T. brucei* mitochondrial proteome has been extensively and rigorously analyzed [17], which allowed the development of high quality multiparametric analyses in bio-informatics [18]. Our starting hypothesis was that, by inhibiting the expression of mitochondrial proteins, we should be able to identify essential proteins associated with this part of the cell cycle in trypanosomatids, defined from cell cycle-specific phenotypes and/or growth reduction. Here, we propose a methodical analysis of the effects of 101 RNAi knockdowns targeting mitochondrial proteins, with the primary aim of determining their potential involvement into cell growth and cell division.

## Results and discussion

### Characteristics of the mitochondrial CDS cohort

This study reports the results of 101 individual RNAi knockdowns performed in procyclic forms (PCF) of *T. brucei* and targeting proteins for which the mitochondrial localization was predicted “with high confidence” in a previous study [17], and for (most of) which the annotation in the *T. brucei* genome database GeneDB [6] was “Hypothetical protein, conserved” at the start of the study. At the time of writing, new annotations have been proposed for a number of these CDSs (See Additional file 1). All the targeted proteins belong to the mitochondrial protein inventory ‘MitoCarta’ [18]). Moreover, all 101 CDSs but two (Tb10.61.1810 and Tb927.7.2990, code name in our study: T217 and T320) were also included in a global approach of “high-throughput phenotyping using parallel sequencing of RNA interference targets” (RIT-seq) developed after the start of our study [8]. Finally, five of the analyzed CDSs were included in a ‘semi-systematic’ RNAi study focused on chromosome 1 of *T. brucei* but utilizing bloodstream forms (BSF) [7].

### Effect of RNAi knockdowns on cell growth

**Figure 1:**
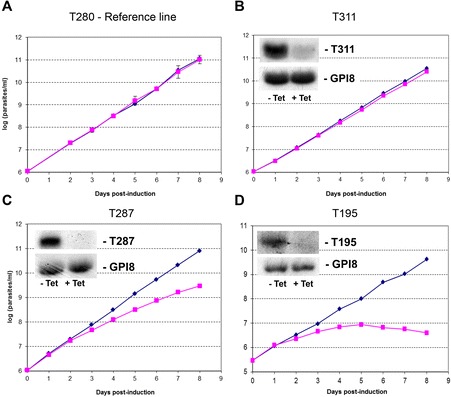
Effect on cell growth rates of RNA interference-based knockdown of mitochondrial proteins in procyclic forms of *Trypanosoma brucei.* Four typical growth curves are shown: **A**: reference cell line (transfected with an ‘empty’ RNAi vector and tetracycline-induced); **B**: no effect; **C**: moderate effect; **D**: severe effect. Procyclic forms were grown in the presence of tetracycline to induce RNAi (+Tet, closed squares), and cell growth was monitored daily for 8 days. Growth curves for uninduced cell lines (−Tet, closed lozenges) are shown for comparison. Insets: silencing was confirmed by Northern blot, using the gene GPI8, constitutively expressed in procyclic trypanosomes, as a loading control; at day2, total RNA was purified and Northern blots were performed to assess levels of mRNA. See definition criteria of the effects on cell growth in Methods.

We used the effect on cell growth at the procyclic stage as a first screen to categorize the results of the 101 RNAi knockdowns. Growth curves were constructed until day 8 post-induction. Growth reductions of at least 50% and 25%, as compared with the uninduced cell line, during the first four days, were defined as severe and moderate effects, respectively. Figure 1 shows three typical cell growth curves for null (B), moderate (C) and severe (D) growth reduction. These criteria, similar to those used in a previous study [7], may appear arbitrary, in particular because the half-life of the targeted proteins is unknown; yet, they allowed us comparing our results with previously published data. In total, 10/101 RNAi experiments yielded a severe reduction of cell growth rates, 29/101 a moderate reduction and 62/101 no reduction. Details of all raw data are presented in Additional file 1. Representative Northern blots of RNAi experiments in each cell growth category are shown in Additional file 2.

Although Subramaniam *et al.* used BSFs when they knocked down 197 CDSs on chromosome 1 [7], they noted that “30% of the chromosome I genes generated a phenotype when targeted by RNAi; most commonly, this affected cell growth, viability, and/or cell cycle progression”. Therefore, the frequencies they observed were not greatly different from the ones reported here. Yet, a detailed comparison of our results with theirs [7] revealed one difference: one RNAi knock-down, out of five examined here, showed a severe growth retardation in BSFs which was not found in our hands in PCFs (T181, Tb927.1.730). The four other ones were concordant in both studies, showing either no effect (n = 3) or a growth defect in the remaining case (Table 1, Additional file 1 and Additional file 2B for Northern blot analysis).

**Table 1:** **Summary of the concordance observed in the frequencies of cell growth phenotypes between our study and previous studies**

**Alsford** ***et al.*** **(2011) [** 8 **]**	**Present study**
No growth reduction (N = 68)	Severe growth reduction (N = 3)^a^
	Moderate growth reduction (N = 22)
	Normal growth (N = 43)
Growth defect (N = 19)	No growth reduction (N = 19)
**Subramaniam** ***et al.*** **(2006) [** 7 **]**	**Present study**
Severe growth retardation^c^ (N = 2)	No growth reduction^d^ (N = 1)^b^
	Severe growth reduction^d^ (N = 1)
No growth reduction^c^ (N = 3)	No growth reduction^d^ (N = 3)

^a^Cell lines T177 (Tb927.9.3640), T194 (Tb927.2.3800), and T271 (Tb927.5.2930); ^b^cell line T181 (Tb927.1.730); ^c^in bloodstream forms; ^d^in procyclic forms.

We then compared our data with those obtained in PCFs using the global approach of RIT-seq [8]: 70 out of our 99 CDSs included in that study were considered not to be associated with growth defects, while 29/99 lead to a growth reduction. These figures appear to be in the same order of magnitude as ours; but this apparent concordance hides significant differences which appear when comparing the results in details (Additional file 1). Indeed, the RIT-seq study reported a cell growth reduction for 19/62 RNAi knockdowns which we classified as having no effect. Conversely, no effect was noted in the RIT-seq study for 22/29 RNAi knockdowns which we classified as having a moderate effect, and for 3/10 which, in our hands, yielded a severe growth reduction. Therefore, a high degree of discrepancy actually exists between both data sets, concerning 44/99 compared proteins. One possible source of these discrepancies is that our PCFs were cultivated *in vitro* for a long time whereas in the RIT-seq study, Alsford *et al.* used induced PCFs directly deriving from BSFs. Discrepancies were significantly lower when we compared our data to those obtained from individual RNAi knockdowns in the literature [9,19-26]. Indeed, this comparison showed a concordance between both datasets in 8/11 cases (see Additional file 1); whereas for three genes (T223/ Tb927.4.1660, mitochondrial carrier protein; T171/ Tb927.1.1160 and T179/ Tb927.1.2990), conflicting results were obtained. As regard Tb927.4.1660, in spite of several attempts, Colasante *et al*. [22] could not obtain a viable null mutant by double knock-out, nor could they obtain it using RNAi knockdown, even in non-induced condition. By generating cell mutants in which the inducible expression of a single c-Myc-fused version of the gene was possible, they could evaluate the effect of the knockdown of this gene, and therefore inferred that it was essential for the parasite. By contrast, in our hands, mutant parasites for this gene were readily obtained (T223); and no growth reduction was observed after tetracycline induction, despite the efficient silencing of the gene (see Northern blots in Additional file 2B).

### Detailed analysis of the major phenotypes associated to a moderate/severe cell growth defect

#### Cell cycle phenotypes

**Figure 2:**
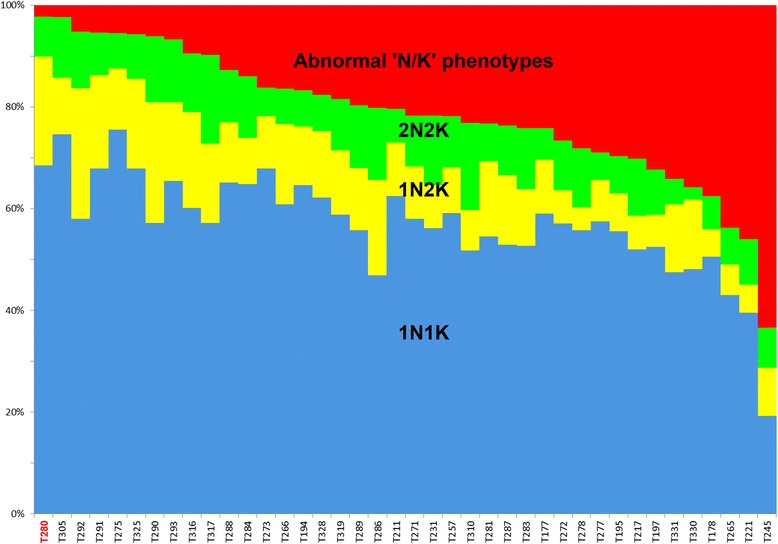
Schematic representation of the different cell cycle phenotypes observed in 39 RNAi mutant cell lines showing a reduction in cell growth. The bars represent the proportions of 1N1K (blue), 1N2K (yellow), 2N2K (green) and “abnormal ‘N/K’ phenotypes” (red), the latter corresponding to phenotypes absent or rare in the reference strain (T280, right-hand side bar). More details about these abnormal phenotypes are given in the additional table and figure (see Figure 2 and Additional file 1). Abscissa : mutant RNAi cell lines code names (see correspondence with CDSs in Additional file 1); ordinate: proportions of the normal N/K phenotypes in %; the cell lines were arbitrarily ordered from left to right according to their proportions of “abnormal ‘N/K’ phenotypes”.

We wished to determine if the cell growth phenotypes could be related with defects in the cell cycle progress. For this, since, in *T. brucei*, cell cycle phases can be recognized by the number and position of DNA-containing organelles, a simple and often used method is the search for abnormal distributions of the numbers of nuclei (N) and kinetoplasts (K) in cells, such as cells containing >2 K and/or >2 N; this disturbs the ‘normal’ distribution pattern, defined by the proportions of 1N1K, 1N2K, and 2N2K cells in the population. For this, cells were stained with DAPI at day 5 post-induction and a detailed examination was performed for all the mutants (39/101) for which a moderate or severe growth reduction was noted (Additional file 1). Surprisingly, all these RNAi knockdown experiments but two (T291 and T325), *i.e.* 37% (37/101) of the CDSs examined in total, yielded abnormal patterns as compared to that of the reference cell line. This is in striking contrast with the data obtained in BSFs by Subramaniam *et al.* [7] when they knocked down all the CDSs of chromosome 1: there, only 6% of the genes were associated to both a growth and a cell cycle defect. The data for these 39 experiments are summarized in Figure 2 and Additional files 1 and 3.

The percentage of 1N1K cells was increased in only two mutant cell lines (T275 and T305), and similar to the reference cell line in three mutants (T273, T291, T325), but lowered in the 34 others. In the mutant line T245 (gene annotated as Mitochondrial processing peptidase, beta subunit, putative metallo-peptidase, Clan ME, Family M16 in GeneDB, but actually characterized by Acestor *et al.* as a component of the respiratome [27]), the reduction in 1N1K cells was particularly drastic, by a 3.6-fold factor as compared with the reference line (*i.e.* 19% *vs.* 68.5% of the cells) (Additional file 1); in this line, we also observed a severe reduction of parasite growth and a large excess of multinucleated cells (37.6% of the cells) and multikinetoplastic cells (22.7%, essentially in multinucleated cells), together with zoids (5.4%) and akinetoplastic cells (4.5%), indicating a complete cytokinesis block in spite of continuing nucleus duplication. 2N1K cells, a rare phenotype in the reference cell line (1.1%), were found in increased proportions in 77% of the RNAi mutants (30/39) (Additional file 1). One of the correlations that might be done is the concomitant increase of zoids (0N1K) and of 2N1K cells that may originate from an abnormal division of normal 2N2K cells. Indeed, in the majority of mutants, the increase of both 0N1K and 2N1K were of the same order of magnitude (see for example T178 and T211). However, in eight cases (T195, T271, T272, T278, T287, T288, T316 and T331), the proportions of 2N1K cells were increased without a corresponding increase in zoids, rather suggesting a kinetoplast replication block.

#### Morphological phenotypes

**Figure 3:**
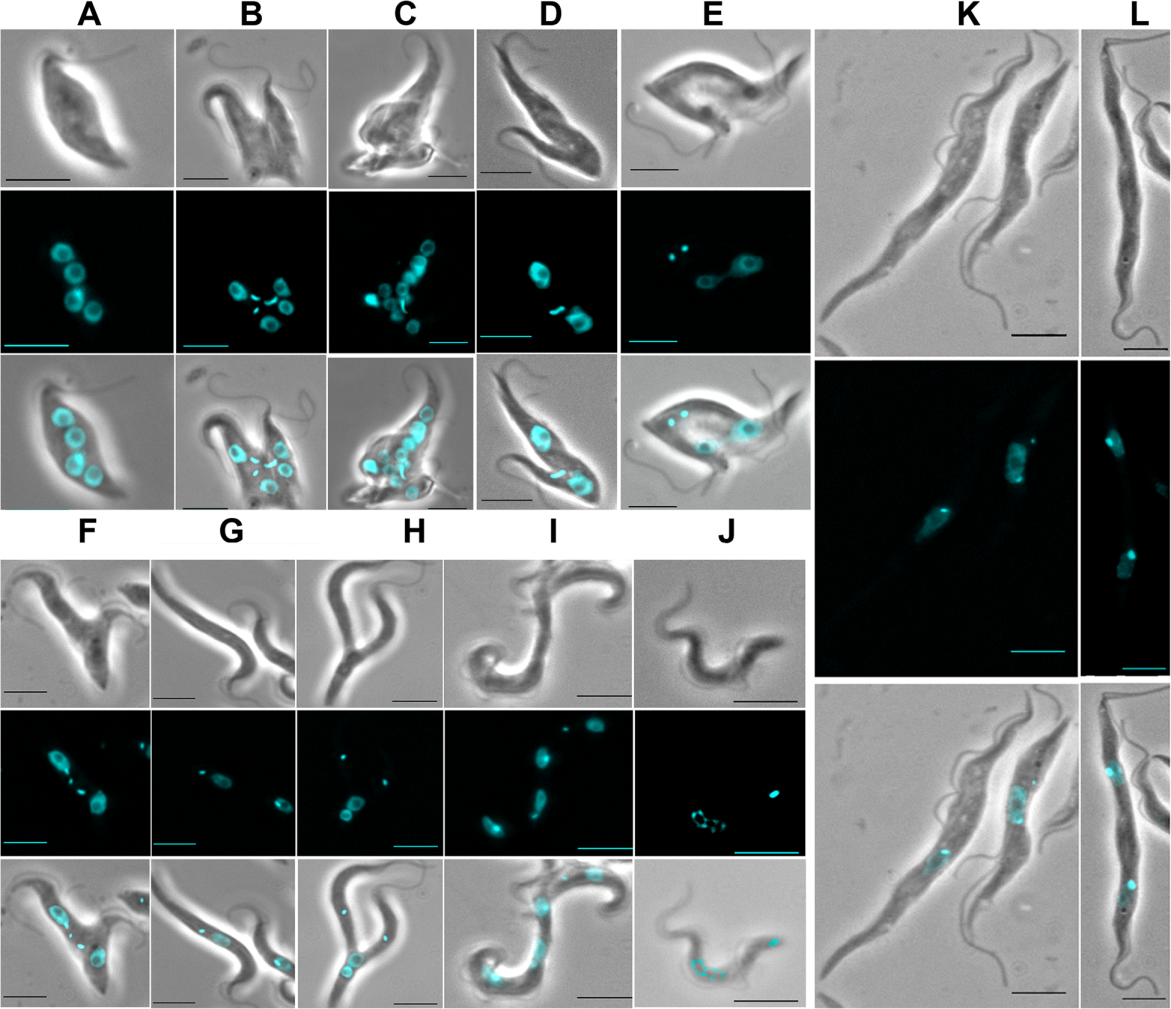
Pleiotropic morphological changes associated with growth defects following RNAi knockdown in *T. brucei.* **A**: Example of a multinucleated cell (observed in the mutant cell line T286). **B**: a multinucleated cells showing aborted cell division and over-replication of the kinetoplasts (T286). **C**: a multinucleated cell showing a complete disorganization of the cell morphology (T286). **D**: a cell with an abnormally segregating kinetoplast (T194). **E**: a dividing cell showing mis-positioning of the kinetoplasts between the dividing nuclei, with impaired cytokinesis (T290). **F**: a dividing cell showing mis-positioning of the kinetoplasts, together with over-replication of the kinetoplasts and impaired cytokinesis (T287). **G**: elongated cells observed in the mutant line T293. **H**: a dividing elongated procyclic form showing abnormal positions of the kinetoplasts (both anterior) and nuclei (both posterior) (T293); a ‘zoid’ might originate from this abnormal division process. **I**: large and elongated multinucleated cells (T317). **J**: fragmented (‘apoptotic-like’) nuclei (T221). **K**: elongated cells observed in the mutant lines T177. **L**: elongated dinucleated cell with impaired cytokinesis (absence of cleavage furrow) with daughter cells still joined at their posterior end (T178). Bar: 5 μm.

Cell growth reduction was often associated with significant cell morphological changes. These morphological phenotypes were most often multinucleated cells (Figure 3A-C) which were significantly increased in 24 cell lines. These cells could have one or two or, more frequently, several kinetoplasts, indicating a cytokinesis block, sometimes leading to a complete disorganization of the cell morphology (Figure 3B-C). Cytokinesis defects (Figure 3D-F) could also be associated with other cell cycle phenotypes, such as abnormally segregating kinetoplasts (Figure 3C-D), and/or mis-positioning of segregated kinetoplasts between the two divided nuclei (Figure 3F). To date, only a single protein, ALBA3, an RNA-binding protein, has been involved in the migration of the nucleus towards the posterior end of the cell in *T. brucei* during cell division [28]. The kinetoplast was found anterior to the nucleus in a very high proportion of cells (22% and 18% of all cells, respectively) in two cell lines, T273 (targeting Tb927.5.3040, the MIX protein [26]), and T325 (targeting Tb927.8.3300, annotated in TriTrypDB as ‘hypothetical conserved’ but purified as a putative mitochondrial LSU ribosomal protein by Zikova et al. [29]): this aberrant positioning (as compared to the expected posterior position) of the kinetoplast was reported after the MIX-RNAi in a previous study [26]. By contrast, the involvement of a putative ribosomal protein in this process is surprising: as mentioned by the authors, this might be explained by the fact that the complexes purified by Zikova et al. [29] are part of a larger supercomplex with additional functions.

In 25/39 cell lines, abnormally segregating kinetoplasts (either grossly enlarged or “thread-like”) were seen, most often in proportions varying from 0.6 to 6.5%. Even when they were in higher proportions, they could not be associated to any specific ‘cell cycle pattern’. The T245 mutant, which was described here above with a gross cytokinesis block, exhibited as much as 20.3% and 19.8% of cells with enlarged and abnormally segregating kinetoplasts, respectively. The whole of these results strongly suggest a blockage of kinetoplast segregation. The corresponding predicted protein was annotated as a the beta subunit of the Mitochondrial processing peptidase (putative metallo-peptidase, Clan ME, Family M16) in GeneDB, but actually characterized by Acestor *et al.* as a component of the respiratome, encoding subunits of the cytochrome bc1 complex [27]. This suggests a largely indirect effect, *i.e.* that the RNAi target is not actually a direct effector of kinetoplast segregation.

Another interesting phenotype affecting the kinetoplast was that of T195, encoding the *bona fide* alpha subunit of the Mitochondrial processing peptidase [21]. T195 displayed a relatively ‘common’ disruption of the ‘N/K’ pattern but an exceptionally high (6.5%) proportion of 0 K cells. A closer examination showed that 74% of the cells had a kinetoplast greatly reduced in size. The RNAi of several proteins closely associated with the kDNA has been previously shown to cause kDNA loss [30-35]. Yet, what appears here as a defect of the kinetoplast maintenance is again likely one indirect effect secondary to the mis-processing of one mitochondrial imported protein among many. Such an indirect role may be illustrated by a protein named TbLOK1 for loss of kDNA: the authors found that the disappearance of the kDNA occurred well after cell division ceased, arguing that the requirement of TbLOK1 for kDNA maintenance is indirect [10].

Cytokinesis was also specifically impaired in two mutant lines (T178 and T310), where long dividing (2N2K) cells were seen still joined at their posterior end in significantly high numbers (Figure 3L); it is noteworthy that, to our knowledge, only one protein, spastin, a microtubule-severing enzyme, has been shown to be functionally involved in this final step of cytokinesis in *T. brucei*, and specifically in bloodstream forms [36].

A number of abnormal morphological phenotypes could not be specifically related to the cell cycle (see Additional file 1). One of these was the presence, in five mutant lines, of particularly long cells (Figure 3H-I), sometimes twice as long as the non-induced cells (*i.e.* ~30 μm; Figure 3K-L), in proportions as high as ~26% in lines T291 and T292, ~20% in two other mutants (T177, T178,) and ~8% in one (T293). In mutant T317, these elongated cells were multinucleated (Figure 3I, T317); but in general these phenotypes could not be correlated with any specific ‘N/K’ phenotype: this suggests that, although a tight association between morphogenesis and the cell cycle in *T. brucei* is well documented [37], the proteins knocked down here are not necessarily involved in a particular step of the cell cycle.

Finally, in T221, the high increase of 2N1K and zoids was associated to the presence of numerous fragmented (‘apoptotic-like’) nuclei (defined in [38]) in 16% of the cells (Figure 3J), as well as a distinctive phenotype consisting in half (44%) of the cells being ‘globular’ with pyknotic nuclei (Additional file 1, Additional file 2A for Northern blot), suggesting a profound disruption of unknown essential functions. The abnormal cell division observed here may then well be an indirect consequence of this more global disorder. Morphologically apoptotic nuclei [38] were seen at a high frequency in only four other lines (T177, T178, T195, and T265; see Additional file 1) but without the ‘globular’ phenotype. However, flow cytometry analysis of the five corresponding cell lines confirmed apoptosis in only two out of five of these (T177 and T178; see Additional file 4).

## Conclusion

Among the 101 mitochondrial protein genes knocked down here by RNAi, 39 could be related to a growth defect of the parasite. This number may seem low, but is in agreement with previous reports [7-9]. Several hypotheses may explain the apparent absence of effect seen in the 62 remaining RNAi knockdowns: (i) there may be a functional complementation by another protein, (ii) they may play a role in another differentiation stage of the parasite, (iii) they may simply be non-essential, at least in the experimental conditions used here.

It came as a surprise to us that almost all 39 proteins for which the expression inhibition yielded a growth reduction appeared to be somehow involved in the cell cycle progress. Clearly, the proportion of such proteins is much higher among mitochondrial proteins (here 37/101, i.e. 37%) than among ‘unsorted’ chromosome 1 proteins (6.6%) [7]. Moreover, there are numerous mitochondrial genes on chromosomes 9–11 of which the knock-down may also induce cell cycle perturbation, but these were not included in this study. However, we believe that a proportion of these proteins is pleiotropic and only indirectly involved in the cell cycle progress: the correct progression of this part of the cell cycle may directly or indirectly depend upon a variety of cell activities, e.g. metabolism, signaling cascades, chaperones, etc. The large variety of morphological changes observed here in the mutants indeed suggests that the disruption of the cell cycle might take place upstream, without the corresponding proteins being directly related to it. Also, although the gene targets were analyzed using Trypanofan, we cannot rule out the possibility that off-target effects occurred, particularly for those genes which can be classified as ‘pleiotropic’.

In total, the ‘semi-systematic’ study presented here opens avenues for the characterization of novel proteins involved in the correct cell cycle progress in *T. brucei*. Our data also show how post-genomics analyses performed on a case-by-case basis may yield discrepancies with global approaches. Finally, they add to the complexity of the picture of cell biology, particularly the cell cycle, of *T. brucei*, characterized by a tight interdependency of multiple biological processes.

## Methods

### Parasites

Procyclic forms of the 29–13 line of *T. brucei* were grown as described [39] at 27°C in SDM-79 (PAA) supplemented with 10% fetal calf serum, 7 μg.mL^−1^ hemin, 30 μg.mL^−1^ hygromycin and 10 μg.mL^−1^ geneticin.

### Selection of targets for RNAi knockdown experiments

We based our work on the results of a proteomic study [17], which are available online [40]. Our selection criteria were: high confidence assignment of the protein to the mitochondrion [17], unknown function at the beginning of the study and sufficient length in order to minimize the risk of off-target effects [41]. Progressing chromosome by chromosome in ascending order, all the genes corresponding to these criteria were systematically targeted, until a total of about 100 genes was attained, giving us sufficient confidence in the proportion of genes associated to a cell cycle phenotype: the genes analyzed were present on chromosome 1 to 8.

### *RNAi knockdown in* T. brucei

To design the primers for the production of RNAi constructs, we used the RNAit–target selection script on the *T. brucei* functional genomics website (TrypanoFAN) [7,42]. Considering oligonucleotide melting temperature and PCR product size, the program is also intended to prevent cross-talk between related gene products in the design of RNAi experiments [43]. The oligonucleotide primers used are all listed in Additional file 5. The different PCR products were cloned into pGEM-Teasy (Promega®) and then into the RNAi vector p2T7tiB/GFP [44]. Transfection and RNAi induction in procyclic forms were performed as described elsewhere [45]. Briefly, 10 μg of linearized plasmid DNA were transfected into 3×10^7^*T. brucei* 29–13 procyclic cells. An exponential transfection protocol was used with 1500 V and 25 μF as parameters with a Bio-Rad® Gene pulser 2 electroporator. Transfectants were grown under selective pressure with 5 μg.mL^−1^ of phleomycin during 8–15 days prior to induction by addition of 1 μg.mL^−1^ of tetracyclin. Growth curves were compared between the induced and non-induced cell lines.

### Definition of the reference line (T280)

For the study of morphological phenotypes, a reference cell line (T280, p2T7-empty) was used for comparison with the mutant cell lines: T280 was transfected with the same RNAi vector containing no insert and then induced in the same manner as the mutant lines. This induced control was considered as the reference cell line; we believe it makes a better control than the uninduced or the wild-type (29–13) cell line of *T. brucei.*

### Northern blots

Total RNAs were extracted with the RNeasy extraction kit (Qiagen) and denatured in a solution of 2.5× MOPS, 9.25% formaldehyde and 50% deionized formamide. RNAs were then incubated 10 min at 65°C and 10 min on ice, before being separated on an agarose gel (1.4% agarose, 6% formaldehyde and MOPS 1×). RNAs were transferred to a nylon membrane and hybridized with a specific probe labeled by random priming with α^32^-dCTP.

### Protocol and criteria for growth reduction definition

The criteria chosen to assess cell growth dynamics used here were as described previously [7]. Briefly, growth of the induced and uninduced culture was followed for up to 8 days after induction, with cell number determined daily using a Z2 Coulter counter (Beckman Coulter®). The numerical data obtained for growth were analyzed according to the following criteria: a defect was recorded if, during the 4 days following induction, cell number in the induced culture was <75% (mild) or <50% (severe) of the uninduced culture for two consecutive days.

### Protocol and criteria for definition of cell cycle defects

At day 5 post-induction, cells were fixed in 4% paraformaldehyde and stained with 4′,6-diamidino2-phenylindole (DAPI), air-dried on microscope immunofluorescence slides, and then analysed on a Zeiss Axioplan 2 microscope with a 100× objective equipped with a Photometrics CoolSNAP charge-coupled device camera (Roper Scientific®) driven by Metamorph Software (Molecular Devices®). The numbers of nuclei (N) and kinetoplasts (K) per cell were then counted in 200 cells, allowing determining the position of these cells in the cell cycle progress. Morphological abnormalities of the nuclei and/or kinetoplasts, as well as of the whole cell, were also recorded. All primary phenotypic data were collected blind. These data were then compared to those obtained for the reference cell line described above (T280), where DAPI analysis indicated ~68% cells with one nucleus (N) and one kinetoplast (K) (1N1K cells), ~20% 1N2K cells, and ~7% 2N2K cells, *i.e.* 95% normal phenotypes. ‘Abnormal’ phenotypes were rare in the reference line: 2.5% 2N1K, 2% zoids (0N1K), <1% multinucleated cells and none for the rest of them. Phenotypes were considered as abnormal in the mutants when they were increased/reduced by a proportion of ± 2.5 SDs, as compared to the induced control (T280). These limits were defined in order to ensure statistical significance to the differences observed. Therefore, an abnormal phenotype was recorded if the following conditions were met: 1N1K <66.1% or >70.9%; 1N2K <13.4% or >29.4%; 2N2K <4.7% or >10.9%; 2N1K >3.8%; multinucleated cells (>2N) >2%; >2N > 2K >0.6%; >2K, 0K and 0N0K >0; and ‘zoids’ (0N) >3.2%.

### DNA content

To determinate the DNA content, a PI staining method was used. For this purpose, cells were washed with PBS, resuspended in 500 μl of iced 70% ethanol, vortexed 1 min and incubated at 4°C. After centrifugation, cells were resuspended in PBS and 10 mg/ml RNAse, placed 20 min at 37°C, centrifuged, incubated 10 – 30 min on ice with 2.5% PI and immediately analysed with a FACSCalibur flow cytometer (Becton Dickinson, San Jose, CA, USA) with the BD CellQuest™ Pro software.

### Phosphatidylserine exposure

Exposed PS was detected on the outer membrane of cells using the Annexin-V-FLUOS staining kit (Roche®). Cells were washed in PBS and incubated for 10–15 min at 4°C with the incubation buffer of the kit. Fluorescence was measured using an FACS analysis.

### Ethics statement

The research presented here does not involve vertebrate (including human subject, human material, human data) or any regulated invertebrate.

## Additional files

Additional file 1:
**Raw data from 101** **RNAi-based** **knockdown experiments in procyclic forms of** ***Trypanosoma brucei.***
Additional file 2:
**Verification of mRNA level reduction by Northern blots.** Representative examples of Northern blots following RNAi experiments leading to a remarkable phenotype (A), contrasting data with the literature (B) and no cell growth reduction (C). GPI8: gene constitutively expressed in procyclic trypanosomes used as a loading control. Additional file 3:
**Proportions of the different abnormal cell cycle ‘N/K’ phenotypes observed in 39 RNAi mutant cell lines showing a reduction in cell growth.** Histograms represent the proportions of the different abnormal cell cycle stages identified by their numbers of nuclei (N) and kinetoplasts (K) (2N1K, >2K, >2N, >2N > 2K, 0N, and 0K) in 39 RNAi mutant cell lines (data from Table 1). The cell lines were arbitrarily ordered from left to right according to their proportions of ‘abnormal N/K phenotypes’ (see Figure 2). Black bar: proportions observed for the reference cell line T280 (in red, left). For the mutant lines, bars are shown in grey when the observed proportions are similar to that of T280 (defined as mean ± 2.5 SDs), and red when they are above these limits. Other: percentage of cells with apoptotic-like nuclei. Additional file 4:
**Flow cytometry analysis of different RNAi mutants displaying ‘N/K’ or ‘apoptotic-like’ phenotypes.** (A). Propidium iodide staining confirmed the ‘N/K’ phenotypes (see Additional file 1) in the four mutants shown here; DNA contents left to the 1C peaks likely correspond to relatively high proportions of zoids; those to the right of the 2C peaks indicate multinucleated (>2N) cells. NI: non-induced; I: induced; D: day. (B). One example of measure of phosphatidylserine exposure through the Annexin V assay in a cell line (T178) displaying an ‘apoptotic-like’ morphological phenotype (fragmented nuclei). The increase of cells in the lower right quadrant classically represents early apoptosis. (C). Summary of the DAPI-staining and flow cytometry data for the five cell lines displaying the most pronounced ‘apoptotic-like’ morphological phenotype. D: day. UR: upper right quadrant; LR: lower right quadrant. Flow cytometry confirmed apoptosis in only two lines: T177 and T178. Additional file 5:
**List of primers used for the production of RNAi constructs.** Restriction site sequences in lower cases, gene specific sequences in upper cases.
