# Supplementary material for: RNA interference screen reveals a high proportion of mitochondrial proteins essential for correct cell cycle progress in Trypanosoma brucei
Source: BMC Genomics. 2015 Apr 15;16(1):297. doi: 10.1186/s12864-015-1505-5 (PMC4445814; doi:10.1186/s12864-015-1505-5)
Supplement: Additional file 2: — Verification of mRNA level reduction by Northern blots. Representative examples of Northern blots following RNAi experiments leading to a remarkable phenotype (A), contrasting data with the literature (B) and no cell growth reduction (C). GPI8: gene constitutively expressed in procyclic trypanosomes used as a loading control. [file 12864_2015_1505_MOESM2_ESM.doc]

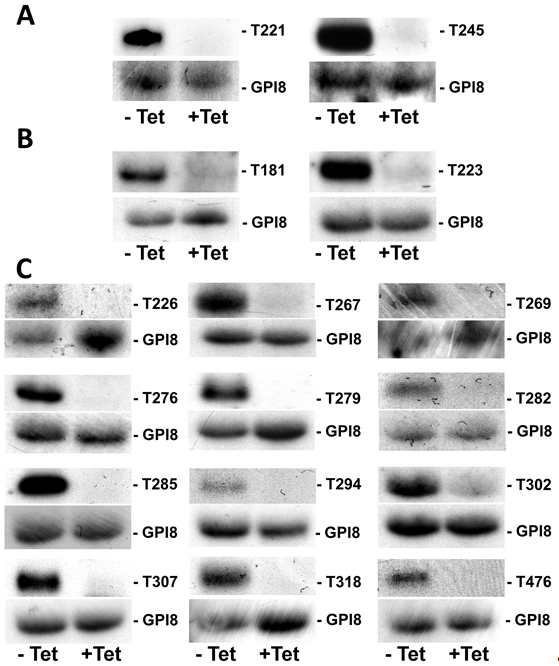


## Additional file 2. Verification of mRNA level reduction by Northern blots.

Representative examples of Northern blots following RNAi experiments leading to a remarkable phenotype (A), contrasting data with the literature (B) and no cell growth reduction (C). GPI8: gene constitutively expressed in procyclic trypanosomes used as a loading control.
