# Supplementary material for: RNA interference screen reveals a high proportion of mitochondrial proteins essential for correct cell cycle progress in Trypanosoma brucei
Source: BMC Genomics. 2015 Apr 15;16(1):297. doi: 10.1186/s12864-015-1505-5 (PMC4445814; doi:10.1186/s12864-015-1505-5)
Supplement: Additional file 3: — Proportions of the different abnormal cell cycle ‘N/K’ phenotypes observed in 39 RNAi mutant cell lines showing a reduction in cell growth. Histograms represent the proportions of the different abnormal cell cycle stages identified by their numbers of nuclei (N) and kinetoplasts (K) (2N1K, >2K, >2N, >2N > 2K, 0N, and 0K) in 39 RNAi mutant cell lines (data from Table 1). The cell lines were arbitrarily ordered from left to right according to their proportions of ‘abnormal N/K phenotypes’ (see Figure 2). Black bar: proportions observed for the reference cell line T280 (in red, left). For the mutant lines, bars are shown in grey when the observed proportions are similar to that of T280 (defined as mean ± 2.5 SDs), and red when they are above these limits. Other: percentage of cells with apoptotic-like nuclei. [file 12864_2015_1505_MOESM3_ESM.doc]

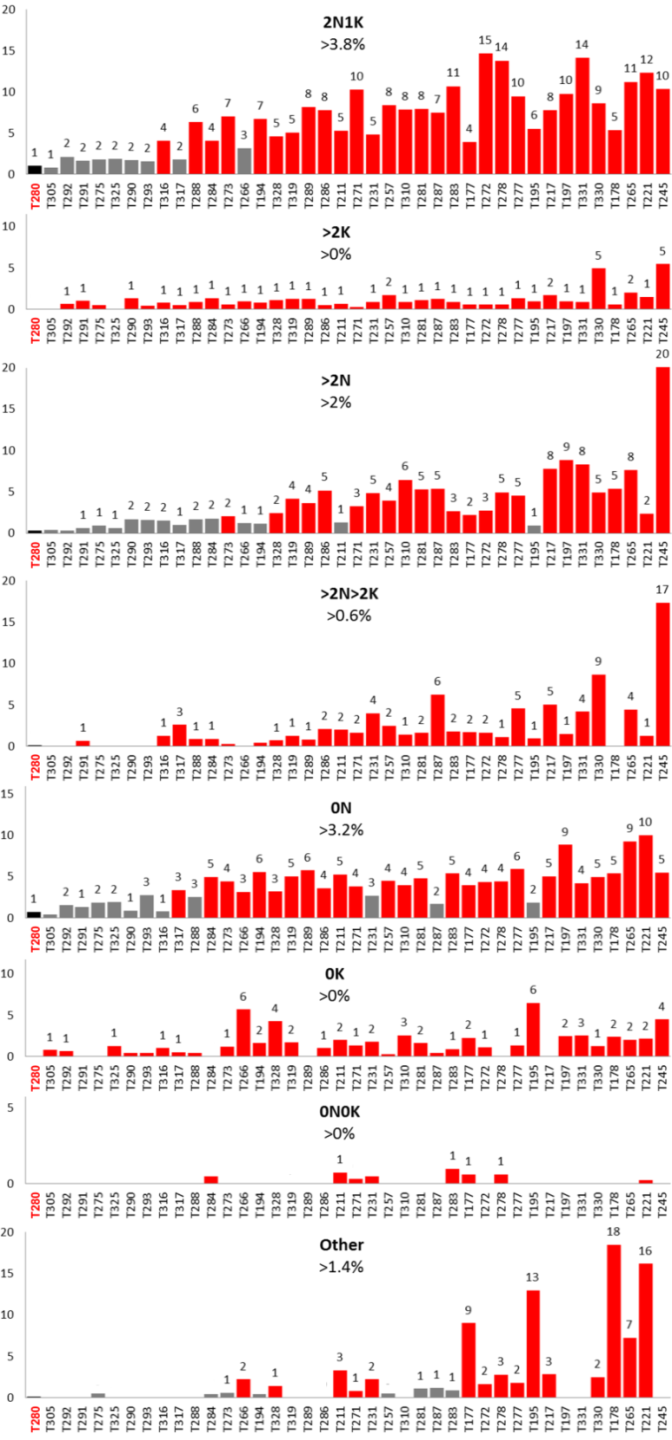


## Additional file 3: Proportions of the different abnormal cell cycle 'N/K' phenotypes observed in 39 RNAi mutant cell lines showing a reduction in cell growth.

Histograms represent the proportions of the different abnormal cell cycle stages identified by their numbers of nuclei (N) and kinetoplasts (K) (2N1K, >2K, >2N, >2N>2K, 0N, and 0K) in 39 RNAi mutant cell lines (data from Table 1). The cell lines were arbitrarily ordered from left to right according to their proportions of 'abnormal N/K phenotypes' (see Figure 2). Black bar: proportions observed for the reference cell line T280 (in red, left). For the mutant lines, bars are shown in grey when the observed proportions are similar to that of T280 (defined as mean ± 2.5 SDs), and red when they are above these limits. Other: percentage of cells with apoptotic-like nuclei.
