# Supplementary material for: RNA interference screen reveals a high proportion of mitochondrial proteins essential for correct cell cycle progress in Trypanosoma brucei
Source: BMC Genomics. 2015 Apr 15;16(1):297. doi: 10.1186/s12864-015-1505-5 (PMC4445814; doi:10.1186/s12864-015-1505-5)
Supplement: Additional file 4: — Flow cytometry analysis of different RNAi mutants displaying ‘N/K’ or ‘apoptotic-like’ phenotypes. (A). Propidium iodide staining confirmed the ‘N/K’ phenotypes (see Additional file 1) in the four mutants shown here; DNA contents left to the 1C peaks likely correspond to relatively high proportions of zoids; those to the right of the 2C peaks indicate multinucleated (>2N) cells. NI: non-induced; I: induced; D: day. (B). One example of measure of phosphatidylserine exposure through the Annexin V assay in a cell line (T178) displaying an ‘apoptotic-like’ morphological phenotype (fragmented nuclei). The increase of cells in the lower right quadrant classically represents early apoptosis. (C). Summary of the DAPI-staining and flow cytometry data for the five cell lines displaying the most pronounced ‘apoptotic-like’ morphological phenotype. D: day. UR: upper right quadrant; LR: lower right quadrant. Flow cytometry confirmed apoptosis in only two lines: T177 and T178. [file 12864_2015_1505_MOESM4_ESM.doc]

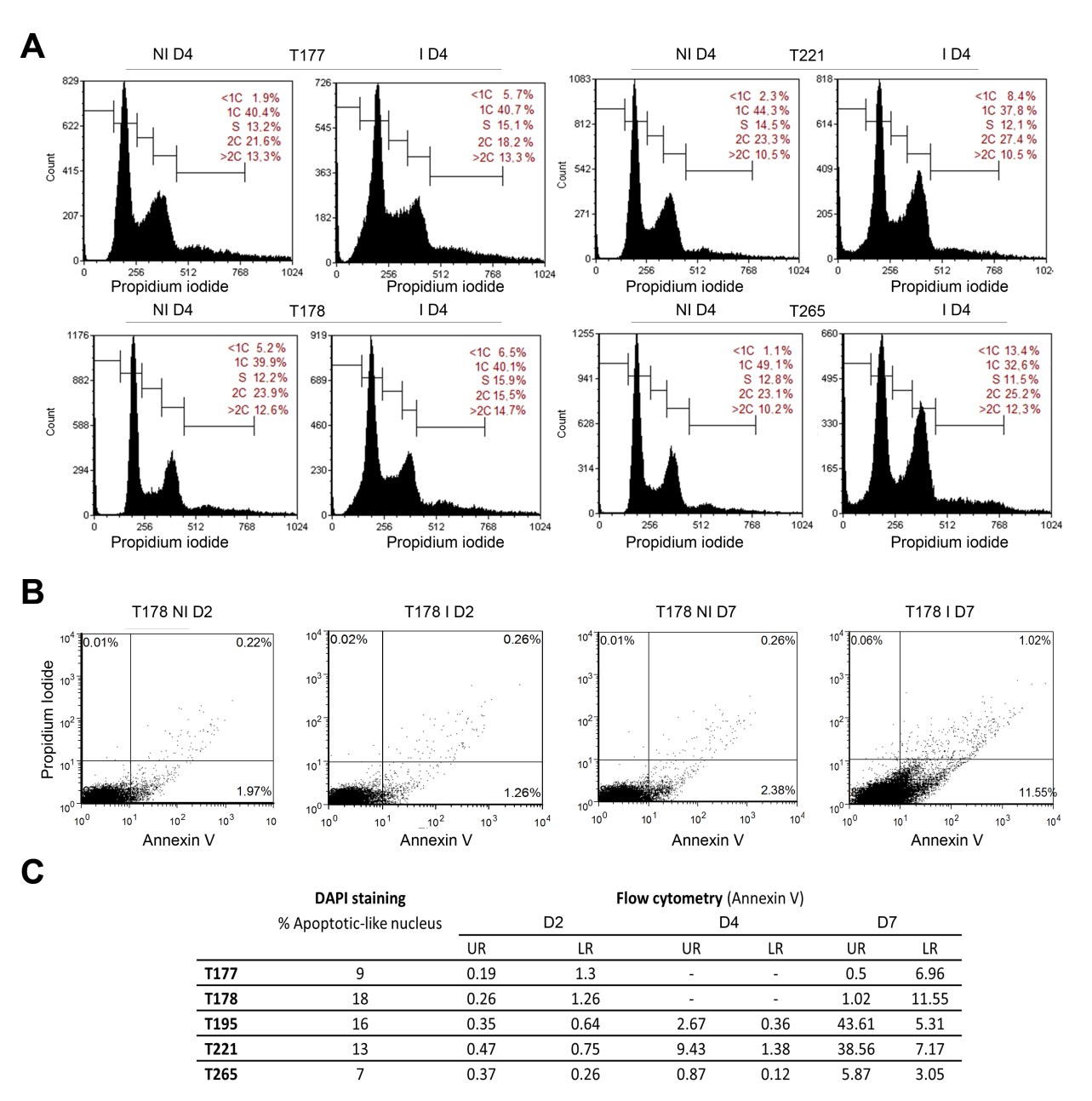


## Additional file 4. Flow cytometry analysis of different RNAi mutants displaying ‘N/K’ or ‘apoptotic-like’ phenotypes.

(A). Propidium iodide staining confirmed the ‘N/K’ phenotypes (see Additional file 1) in the four mutants shown here; DNA contents left to the 1C peaks likely correspond to relatively high proportions of zoids; those to the right of the 2C peaks indicate multinucleated (>2N) cells. NI: non-induced; I: induced; D: day. (B). One example of measure of phosphatidylserine exposure through the Annexin V assay in a cell line (T178) displaying an ‘apoptotic-like’ morphological phenotype (fragmented nuclei). The increase of cells in the lower right quadrant classically represents early apoptosis. (C). Summary of the DAPI-staining and flow cytometry data for the five cell lines displaying the most pronounced ‘apoptotic-like’ morphological phenotype. D: day. UR: upper right quadrant; LR: lower right quadrant. Flow cytometry confirmed apoptosis in only two lines: T177 and T178.
