# Supplementary material for: RNA interference screen reveals a high proportion of mitochondrial proteins essential for correct cell cycle progress in Trypanosoma brucei
Source: BMC Genomics. 2015 Apr 15;16(1):297. doi: 10.1186/s12864-015-1505-5 (PMC4445814; doi:10.1186/s12864-015-1505-5)
Supplement: Additional file 1: — Raw data from 101 RNAi-based knockdown experiments in procyclic forms of Trypanosoma brucei. [file 12864_2015_1505_MOESM1_ESM.zip › 1270723661124619_add2.doc]

## Additional file 1. Raw data from 101 RNAi-based knockdown experiments in procyclic forms of *Trypanosoma brucei*.

All the CDSs targeted in this study are listed by numerical order of the construct code name (except the reference cell line T280, see Materials & Methods, top grey line). The "GeneDB annotation" column differentiates them according to their annotation in GeneDB and boxes are color-coded accordingly: ‘Hypothetical protein, conserved’: orange boxes; CDSs for which a function was inferred by sequence homology: yellow boxes; CDSs for which a function has been established from experimental data: red boxes. Effects on cell growth reduction were categorized as moderate (blue boxes), and major (dark blue boxes). The phenotypes analyzed were essentially based upon the numbering of nuclei (N) and kinetoplasts (K); the different categories of cells: 1NK, 1N2K, 2N2K, 2N1K, >2K, >2N, >2N>2K, 0N, 0K and "other" are mutually exclusive. The percentages observed for each category in each mutant are indicated in the boxes: boxes are light grey, grey or dark grey, depending whether the percentages are lower, similar or higher, respectively, to those observed in the reference cell line T280. See Methods for the definitions of "lower", "similar" or higher". The "Abnormal morphological phenotype" column shows notable morphological abnormalities found in the 39 lines for which a growth defect was recorded (% of cells analysed). BSF: bloodstream form; PCF: procyclic form; Mitocarta: Data obtained by Zhang et al. [ref. 1 below]. Data obtained by "RIT-seq" [ref. 2 below] are shown on the right hand side for comparison; the discrepancies observed about the effects of each RNAi knockdown on cell growth (in PCF only) are indicated by red boxes, whereas concordance is shown by green boxes. [1-29]: references listed below. In the list of references, studies which include RNAi targeting of individual genes data are highlighted in yellow; the references in bold or in bold and underlined present consistent (n=8) or discrepant (n=3) results, respectively, with the present study. 'Not included' means 'not included in the corresponding study' ([1] or [2]). *T245 was actually characterized as a component of the *T. brucei* respiratome [7]. **Other: percentage of cells with fragmented nuclei.

**References for this table**

1.	Zhang X, Cui J, Nilsson D, Gunasekera K, Chanfon A, Song X, Wang H, Xu Y, Ochsenreiter T: **The *Trypanosoma brucei* MitoCarta and its regulation and splicing pattern during development**. *Nucleic Acids Res* 2010, **38**(21):7378-7387.

2.	Alsford S, Turner DJ, Obado SO, Sanchez-Flores A, Glover L, Berriman M, Hertz-Fowler C, Horn D: **High-throughput phenotyping using parallel sequencing of RNA interference targets in the African trypanosome**. *Genome Res* 2011, **21**(6):915-924.

3.	Siegel TN, Hekstra DR, Wang X, Dewell S, Cross GA: **Genome-wide analysis of mRNA abundance in two life-cycle stages of *Trypanosoma brucei* and identification of splicing and polyadenylation sites**. *Nucleic Acids Res* 2010, **38**(15):4946-4957.

4.	Vertommen D, Van Roy J, Szikora JP, Rider MH, Michels PA, Opperdoes FR: **Differential expression of glycosomal and mitochondrial proteins in the two major life-cycle stages of *Trypanosoma brucei***. *Mol Biochem Parasitol* 2008, **158**(2):189-201.

5.	Subramaniam C, Veazey P, Redmond S, Hayes-Sinclair J, Chambers E, Carrington M, Gull K, Matthews K, Horn D, Field MC: **Chromosome-wide analysis of gene function by RNA interference in the african trypanosome**. *Eukaryot Cell* 2006, **5**(9):1539-1549.

6.	Panigrahi AK, Zikova A, Dalley RA, Acestor N, Ogata Y, Anupama A, Myler PJ, Stuart KD: **Mitochondrial complexes in *Trypanosoma brucei*: a novel complex and a unique oxidoreductase complex**. *Mol Cell Proteomics* 2008, **7**(3):534-545.

7.	Acestor N, Zikova A, Dalley RA, Anupama A, Panigrahi AK, Stuart KD: ***Trypanosoma brucei* mitochondrial respiratome: composition and organization in procyclic form**. *Mol Cell Proteomics* 2011, **10**(9):M110.006908.

8.	Ammerman ML, Downey KM, Hashimi H, Fisk JC, Tomasello DL, Faktorova D, Kafkova L, King T, Lukes J, Read LK: **Architecture of the trypanosome RNA editing accessory complex, MRB1**. *Nucleic Acids Res* 2012, **40**(12):5637-5650.

9.	Colasante C, Pena Diaz P, Clayton C, Voncken F: **Mitochondrial carrier family inventory of *Trypanosoma brucei* brucei: Identification, expression and subcellular localisation**. *Mol Biochem Parasitol* 2009, **167**(2):104-117.

10.	Zikova A, Schnaufer A, Dalley RA, Panigrahi AK, Stuart KD: **The F(0)F(1)-ATP synthase complex contains novel subunits and is essential for procyclic *Trypanosoma brucei***. *PLoS Pathog* 2009, **5**(5):e1000436.

11.	Hashimi H, Cicova Z, Novotna L, Wen YZ, Lukes J: **Kinetoplastid guide RNA biogenesis is dependent on subunits of the mitochondrial RNA binding complex 1 and mitochondrial RNA polymerase**. *RNA* 2009, **15**(4):588-599.

12.	Desy S, Schneider A, Mani J: ***Trypanosoma brucei* has a canonical mitochondrial processing peptidase**. *Mol Biochem Parasitol* 2012, **185**(2):161-164.

13.	Zikova A, Panigrahi AK, Dalley RA, Acestor N, Anupama A, Ogata Y, Myler PJ, Stuart K: ***Trypanosoma brucei* mitochondrial ribosomes: affinity purification and component identification by mass spectrometry**. *Mol Cell Proteomics* 2008, **7**(7):1286-1296.

14.	Nilsson D, Gunasekera K, Mani J, Osteras M, Farinelli L, Baerlocher L, Roditi I, Ochsenreiter T: **Spliced leader trapping reveals widespread alternative splicing patterns in the highly dynamic transcriptome of *Trypanosoma brucei***. *PLoS Pathog* 2010, **6**(8):e1001037.

15.	Jones A, Faldas A, Foucher A, Hunt E, Tait A, Wastling JM, Turner CM: **Visualisation and analysis of proteomic data from the procyclic form of Trypanosoma brucei**. *Proteomics* 2006, **6**(1):259-267.

16.	Zikova A, Panigrahi AK, Uboldi AD, Dalley RA, Handman E, Stuart K: **Structural and functional association of *Trypanosoma brucei* MIX protein with**. *Eukaryot Cell* 2008, **7**(11):1994-2003.

17.	Aphasizheva I, Maslov D, Wang X, Huang L, Aphasizhev R: **Pentatricopeptide repeat proteins stimulate mRNA adenylation/uridylation to activate mitochondrial translation in trypanosomes**. *Mol Cell* 2011, **42**(1):106-117.

18.	Acestor N, Panigrahi AK, Ogata Y, Anupama A, Stuart KD: **Protein composition of *Trypanosoma brucei* mitochondrial membranes**. *Proteomics* 2009, **9**(24):5497-5508.

19.	Stephens JL, Lee SH, Paul KS, Englund PT: **Mitochondrial fatty acid synthesis in *Trypanosoma brucei***. *J Biol Chem* 2007, **282**(7):4427-4436.

20.	Bridges DJ, Pitt AR, Hanrahan O, Brennan K, Voorheis HP, Herzyk P, de Koning HP, Burchmore RJ: **Characterisation of the plasma membrane subproteome of bloodstream form *Trypanosoma brucei***. *Proteomics* 2008, **8**(1):83-99.

21.	Colasante C, Alibu VP, Kirchberger S, Tjaden J, Clayton C, Voncken F: **Characterization and developmentally regulated localization of the mitochondrial carrier protein homologue MCP6 from *Trypanosoma brucei***. *Eukaryot Cell* 2006, **5**(8):1194-1205.

22.	Richterova L, Vavrova Z, Lukes J: **DEAD-box RNA helicase is dispensable for mitochondrial translation in *Trypanosoma brucei***. *Exp Parasitol* 2011, **127**(1):300-303.

23.	Kafkova L, Ammerman ML, Faktorova D, Fisk JC, Zimmer SL, Sobotka R, Read LK, Lukes J, Hashimi H: **Functional characterization of two paralogs that are novel RNA binding proteins influencing mitochondrial transcripts of *Trypanosoma brucei***. *RNA* 2012, **18**(10):1846-1861.

24.	Saxowsky TT, Choudhary G, Klingbeil MM, Englund PT: **Trypanosoma brucei has two distinct mitochondrial DNA polymerase beta enzymes**. *J Biol Chem* 2003, **278**(49):49095-49101.

25.	Broadhead R, Dawe HR, Farr H, Griffiths S, Hart SR, Portman N, Shaw MK, Ginger ML, Gaskell SJ, McKean PG *et al*: **Flagellar motility is required for the viability of the bloodstream trypanosome**. *Nature* 2006, **440**(7081):224-227.

26.	Ammerman ML, Hashimi H, Novotna L, Cicova Z, McEvoy SM, Lukes J, Read LK: **MRB3010 is a core component of the MRB1 complex that facilitates an early step of the kinetoplastid RNA editing process**. *Rna* 2011, **17**(5):865-877.

27.	Monnerat S, Clucas C, Brown E, Mottram JC, Hammarton TC: **Searching for novel cell cycle regulators in *Trypanosoma brucei* with an RNA interference screen**. *BMC Res Notes* 2009, **2**:46.

28.	Weng J, Aphasizheva I, Etheridge RD, Huang L, Wang X, Falick AM, Aphasizhev R: **Guide RNA-binding complex from mitochondria of trypanosomatids**. *Mol Cell* 2008, **32**(2):198-209.

29.	Pusnik M, Small I, Read LK, Fabbro T, Schneider A: **Pentatricopeptide repeat proteins in *Trypanosoma brucei* function in mitochondrial ribosomes**. *Mol Cell Biol* 2007, **27**(19):6876-6888

.
